# The scientific publication of the Memórias do Instituto Oswaldo Cruz (1909-2020): a history of contribution to the biomedical sciences

**DOI:** 10.1590/0074-02760210376

**Published:** 2022-06-13

**Authors:** Fabio Batista Mota, Luiza Amara Maciel Braga, Bernardo Pereira Cabral, Renato Matos Lopes, Luiz Anastácio Alves

**Affiliations:** 1 Fundação Oswaldo Cruz-Fiocruz, Instituto Oswaldo Cruz, Laboratório de Comunicação Celular, Rio de Janeiro, RJ, Brasil; 2 Universidade Federal Fluminense, Faculdade de Economia, Niteroi, RJ, Brasil; 3 Universidade Federal da Bahia, Departamento de Economia, Salvador, BA, Brasil

**Keywords:** Memórias do Instituto Oswaldo Cruz, journal article, research topics, infectious and parasitic diseases, bibliometrics, social network analysis

## Abstract

**BACKGROUND:**

The Memórias do Instituto Oswaldo Cruz (MIOC) is one of the first scientific journals created in Brazil and currently one of the most important biomedical journals in South America. Knowledge of the main themes disseminated over time and its main contributors can contribute towards a better understanding of its trajectory and future.

**OBJECTIVES:**

Map the journal’s scientific publication between 1909 and 2020.

**METHODS:**

Data from three scientific databases was combined, alongside bibliometrics and network analysis to analyse publication records between 1909 and 2020.

**FINDINGS:**

Publications increased substantially since the 1980s. The main publishing organisations are Brazilian. Excluding Brazil, the main publishing countries are the USA, Argentina, and Colombia. During the entire investigated period, the main themes refer to Chagas disease, schistosomiasis, and Leishmaniasis. During some periods, publications followed disease outbreaks in Brazil (*e.g.*, dengue fever and yellow fever).

**MAIN CONCLUSIONS:**

Since its foundation in 1909, the MIOC has focused on infectious and parasitic diseases. The editorial changes implemented from the 1980s onwards led MIOC to a relevant growth concerning annual publications and its transformation into an important communication vehicle for researchers from several Brazilian organisations besides Fiocruz, as well as organisations from other countries, especially within Latin America.

Scientific journals have comprised essential cornerstones for science development since the creation of first formal journals in 1665, namely Les Journal des Sçavans (now Le Journal des Savants) and Philosophical Transactions.^1^ ^,^ ^2^ ^,^ ^3^ The Memórias do Instituto Oswaldo Cruz (Oswaldo Cruz Institute Memories) (MIOC) was one of the first Brazilian scientific journals, and certainly the most important one in the first decades of the 20th century.^4^

The origin of the MIOC goes back to Brazilian Decree Nº 1,802, of December 12, 1907, which, besides creating the Instituto de Pathologia Experimental de Manguinhos (Manguinhos Institute of Experimental Pathology) - renamed Instituto Oswaldo Cruz (Oswaldo Cruz Institute) (IOC) in 1908 -, determined that its studies should be “published, as Memories, as the experiences are confirmed”.^5^ Since its foundation, the MIOC has received institutional and financial support from its host Institution - the IOC as well as from the Oswaldo Cruz Foundation since 1970. This support has allowed the MIOC to be freely accessible online since the early 1990s and, more recently, has also facilitated the adoption of a full open access publishing policy, including no article processing charges (APCs). This open access feature has contributed to the MIOC becoming highest impact factor (IF) journal among Latin American scientific journals.^6^ Currently, the MIOC is indexed in 19 scientific bases, including the Scientific Electronic Library Online (SciELO), PubMed, and Web of Science Core Collection (WoS) (SciELO: scielo.br/journal/mioc/about/).

The first edition of the MIOC, released in April 1909, was credited to efforts made by the physician and scientist Oswaldo Cruz.^7^ Oswaldo Cruz is, to this day, considered the greatest public health figure in Brazil. Among other achievements, Oswaldo Cruz is credited with controlling smallpox and yellow fever outbreaks in Rio de Janeiro in the early 1900s and creating the first biomedical graduate program in Brazil, the Applied Manguinhos Course.^8^

Long history journals can comprise a way to understand changes that take place in science, technology, and society through their disseminated content over time. Indeed several studies have sought to map the knowledge disseminated in relevant journals founded decades ago.^9^ ^-^ ^16^ Although in the past this was carried out by manually reading available texts,^11^ ^,^ ^14^ bibliometrics and social network analysis (SNA) tools have been increasingly adopted in recent decades.^9^ ^,^ ^10^ ^,^ ^12^ ^,^ ^13^ ^,^ ^15^ ^,^ ^16^

Bibliometrics employs scientific publication metadata and data/text mining software to analyse frequencies or co-occurrences and produce descriptive information concerning a given variable or combination of variables. This field can, for example, identify and produce information concerning the most publishing organisations and countries in a given field using author affiliation data, as well as map the evolution over time of a certain research subject using Medical Subject Headings (MeSH) terms or author keywords. In turn, SNA employs metadata from scientific publications and network analysis software to analyse statistical relationships (*e.g.*, co-occurrences) among the same or different variables. As in the previous example, SNA cannot only identify the most collaborative organisations and countries by analysing co-occurrences among author affiliations, but also measure the relevance of their relationships through a variety of metrics, such as weighted degree, closeness centrality, and betweenness centrality.

The MIOC has been disseminating scientific knowledge generated not only at the IOC but in many organisations worldwide for over 100 years. The prominent position this journal holds in the history of science in Brazil is, therefore, very clear. Previous studies have analysed MIOC publications, but only qualitatively or bibliometrically, without covering the research subjects disseminated since its foundation.^4^ ^,^ ^17^ ^,^ ^18^ ^,^ ^19^ This study addresses this gap by mapping the MIOC’s scientific publication from 1909 to 2020 to identify the main research topics it has disseminated over time, as well as its main collaborating organisations. To this end, data from SciELO, PubMed, and WoS were combined, and bibliometric and SNA were performed to assess articles published by the MIOC from its first edition up to 2020.

## MATERIALS AND METHODS

*Search strategies and data collection* - Records of articles and review articles published by the MIOC were obtained from the SciELO, PubMed, and WoS databases. The search strategies are presented in Table I. The search and data collection were performed in May 2020.

**TABLE I:** Search strategies used to find articles and review articles published by the Memórias do Instituto Oswaldo Cruz (MIOC)

Database	Main query / Publication title*	Filter/Document type	Period	Results
SciELO	(ta:(memórias do instituto oswaldo cruz))	Article, review article	All years (1909-2020)	7,387
PubMed	“Memórias do Instituto Oswaldo Cruz”(Journal)	Journal article, review	All years (1945-2020)	6,117
WoS	so = (memorias do instituto oswaldo cruz)	Article, review	All years (1945-2020)	5,479

*The field tags ‘ta’, ‘journal’, and ‘so’ refer to the journal titles.

**Fig. 1:**
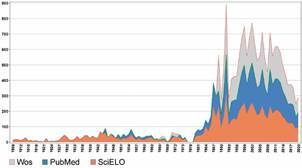
Memórias do Instituto Oswaldo Cruz (MIOC) publication distribution over time by database (1909-2020).

*Data treatment and analysis* - The metadata regarding the publication records was imported into the data/text mining software VantagePoint, for data treatment and subsequent bibliometric analysis. A dataset was created for each database, and, after analysing publication record distribution over time (Fig. 1), we choose to create three sub-datasets comprising different periods for each database with no intersection between them. This is because (i) only the SciELO database covers the period from 1909-1944, (ii) the WoS’ first MIOC record is 1966 and there are no records for 1974-1988, and (iii) from 1989 onwards PubMed contains slightly fewer records than WoS. Thus, the SciELO sub-dataset covers 1909-1944, PubMed, 1945-1988, and WoS, 1989-2020. We imported fields common to all databases and relevant for the analysis into these sub-datasets, referring to titles, abstracts, author names, author affiliations, countries, and publication year. For the WoS and PubMed sub-datasets, we also import the DOI and PubMed ID fields, while also importing the MeSH Terms (Top Level) field for the PubMed sub-dataset only.

All three sub-datasets were then combined using the VantagePoint Dataset Fusion. This new dataset (main dataset) covers the period from 1909-2020. We proceeded in two ways to fill the record gap in the MeSH Terms (Top Level) field, a controlled vocabulary that indexes PubMed publications (MeSH: ncbi.nlm.nih.gov/mesh/). First, we imported the MeSH Terms (Top Level) from 1989 to 2020 from the PubMed sub-dataset using Record Fusion from VantagePoint. Next, since the first record of a MIOC publication in PubMed is from 1945, we generated a proxy for MeSH Terms (Top Level) for the 1909-1944 period, by reading the 752 titles indexed in SciELO obtained for this period and selecting their keywords. These keywords were then used in the MeSH search. The top MeSH terms corresponding to the keywords of the titles were then entered as MeSH Terms (Top Level) in the main dataset. In total, we obtained 311 MeSH terms distributed among the 752 MIOC publications indexed in SciELO from 1909 to 1944.

Information concerning countries and organisations refers to author affiliation (author affiliations include the first author, co-authors, and corresponding authors). For example, an article by an author affiliated with Harvard University will be assigned to the country United States of America and the organisation Harvard University. An article is assigned to all countries and organisations to which its authors are affiliated. Thus, an article can be counted for multiple countries and organisations. Employing VantagePoint, we cleaned and standardised country and organisation records using the List Cleanup tool (fuzzy logic general) along with manual cleaning. For countries, after cleaning and standardisation, we used the VantagePoint Group using Thesaurus tool and the thesaurus of Regions to assign countries to their geographic regions. Information of publication countries is related to author affiliations and begin appearing in our main dataset from 1976. However, the data is more consistent from 1989 onwards (the WoS period of the main dataset). Thus, we employed the 1989-2020 period for the data analysis. The same was performed for the organisation data.

Concerning the MeSH Terms (Top Level), the VantagePoint’s tool Group using Thesaurus and the thesaurus MeSH to Semantic Type were used to classify the terms into groups comprising the same semantic meaning. After this process, the 5,064 MeSH Terms (Top Level) were classified into 120 semantic groups, of which four were selected for further analysis as the terms within them were considered representative of MIOC’s publications, namely virus, bacterium, eukaryote, and disease or syndrome. Then, the Jupyter Notebook and the Python seaborn, word cloud, and matplotlib libraries were employed to generate time evolution word clouds of the top 50 terms within each semantic group. The size of the terms in each word cloud reflects the frequency with which they appear in a given period.

Next, we selected the Disease or Syndrome group for further treatment to depict the evolution of the most frequent diseases or syndromes over time within MIOC publications. To this end, the VantagePoint’s List Cleanup tool (fuzzy logic general) along with MeSH were employed to analyse MeSH Terms (Top Level) records with a frequency of over 50. Generic terms such as acute disease, endemic diseases, chronic diseases were excluded from the analysis, while terms under 50 associated to terms presenting a frequency above 50 were grouped. For example, the terms AIDS-related complex, acquired immunodeficiency syndrome, AIDS-related opportunistic infections, were grouped into human immunodeficiency virus (HIV) infections, which, in the MeSH hierarchy, contains all these terms.

**Fig. 2:**
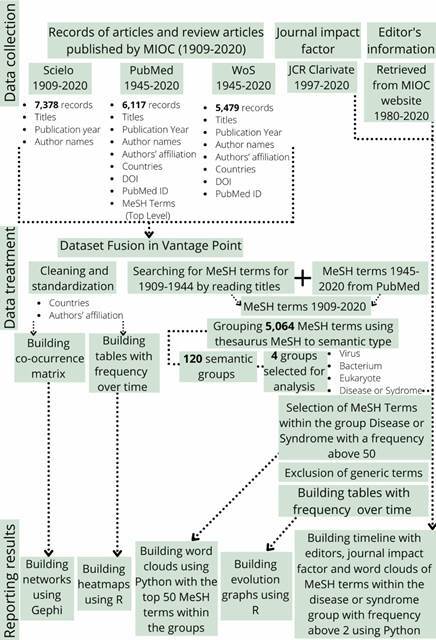
method flowchart.

VantagePoint was also used to create co-occurrence matrices comprising countries and organisations. These matrices were then imported into the Gephi 0.9.2 network analysis software, where networks were plotted and the metrics used to perform the network analysis were generated, namely degree (D), weighted degree (WD), closeness centrality (CC), betweenness centrality (BC), and eigenvector centrality (EC). The ForceAtlas2 algorithm^20^ provided the layouts of all networks. Except for the word cloud Figure, all other figures were generated using RStudio and the ggplot2 and egg R packages. MIOC IF data were obtained from Clarivate Journal Citation Reports™ (Clarivate: clarivate.com/webofsciencegroup/solutions/journal-citation-reports/) and the names and period data of its Editors-in-Chief were obtained from the journal’s website (MIOC: memorias.ioc.fiocruz.br/memorias-board). Fig. 2 presented the applied method flowchart.

*Network metrics* - The degree comprises the number of connections (edges) of a certain node. If a node is connected to three other nodes, its degree will be three. The node with the highest degree is the most interconnected and will disseminate information directly (without intermediation) to the highest number of network nodes.^21^ The weighted degree is the sum of these connections, ponderated by the individual weight of the connections.^21^ The connection weight is the sum of the co-occurrences between the nodes.^21^ A node with three connections, two with weight 1 and one with weight 2, will have a weighted degree of four. The weighted degree is used when not only the number of connections but also the strength (weight) in which they occur are important.^21^ The closeness centrality quantifies how close a node is to all the other nodes by calculating the shortest paths (distance) between all network nodes. The distance between two nodes is the number of edges between them.^22^ The node that presents the shortest average path to reach all other nodes exhibits the highest closeness centrality. Nodes with the highest closeness centrality are closest to the others.^23^ ^,^ ^24^ The closeness centrality is used to identify nodes that will quickly spread information throughout the network.^25^ Betweenness centrality determines how often a node appears in the shortest paths between network nodes, serving as a knowledge bridge between nodes with no connection to each other.^21^ ^,^ ^24^ ^,^ ^26^ Betweenness centrality identifies nodes connected to isolated nodes, and without their presence the connection density of the network will decrease.^26^ The Eigenvector centrality assesses the number of connections a node presents with central nodes.^22^ ^,^ ^23^ ^,^ ^27^ The node with the highest eigenvector centrality exhibits the highest number of connections to central nodes.^22^ The Eigenvector centrality finds the nodes that, by connecting to other central nodes, are the most influent regarding knowledge spreading throughout the network.^22^

*Study limitations* - As this study mapped articles published by a scientific journal, no main subject of analysis (such as a given disease, research method, or technology) is indicated herein. At the same time several subjects were published by the MIOC over 100 years. As we attempted to make sense of a large volume of data with frequencies related to a diversity of subjects displaying weak co-occurrences, the absence of a main subject posed additional difficulties to the bibliometric and network analysis. Thus, the mapping is limited to a certain extent to an overview of the main research subjects disseminated by MIOC over time.

While the author’s keywords are often subjective, non-standardised, and non-related to an index (requiring record cleaning and standardisation), MeSH is a hierarchical-controlled vocabulary thesaurus. Besides being well known in the academic community, the use of MeSH terms avoids biases that may be introduced when performing record cleaning and standardisation. Because of this, MeSH Terms (Top Level) were employed instead of author keywords to classify MIOC publications. The absence of a main subject of analysis, however, led us to group MeSH Terms (Top Level) employing a semantic MeSH term thesaurus. Despite a certain degree of generalisation, the use of this thesaurus allowed us to classify widely dispersed terms into groups with common meanings. On the one hand, generalisations may refer to the automatic inclusion of generic terms into a given semantic group, such as Disease or Infections into the Disease or Syndrome group. On the other hand, it may refer to a very broad, unspecific semantic group, such as Eukaryote, one of the three domains of life, along with Bacteria and Archaea. Nevertheless, as depicted in the word clouds, the MeSH terms within the semantic group Eukaryote offer a good content of the main research subjects published by the MIOC.

## RESULTS

**Fig. 3:**
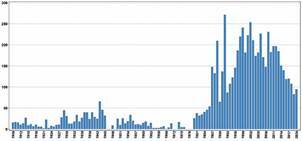
Memórias do Instituto Oswaldo Cruz (MIOC) publication distribution over time (1909-2020).

Fig. 3 indicates MIOC publication distribution over time from 1909 to 2020. From 1909 to 1985, the MIOC published less than 50 articles per year (except for 1943, with 66 articles). No records of MIOC publications for 1946, 1947, 1949, 1972, 1973, 1977-1979 area available. The absence of records for part of the 1940s and early 1970s is due to the non-existence of these data in PubMed, while the absence in the late 1970s is related to the military dictatorship in Brazil and will be explained in the discussion section. From 1987 to 2018, except for 1990 (n = 50) and 1993 (n = 87), over a hundred articles were published per year, with a peak of 272 articles published in 1992. From 2002 onwards there seems to be a downward trend in the number of annual publications, below one hundred since 2019.

From 1989 to 2020, 106 countries published 4,859 publications. Brazil accounts for 69.91% of total publications, followed by the United States of America (USA) (9.22%), Argentina (6.36%), and France (4.45%). Even when excluding Brazil, most MIOC-publishing countries are from South America (37.33% of 2,108 publications). Europe ranks second (33.87%), followed by North America (22.77%), and Central America and the Caribbean (10.67%). Together, Asia, Africa, the Middle East, and Oceania total 15.32%. One should note that, due to country co-occurrences (*i.e.*, countries that publish together), the percentages of countries and regions do not match 100%).

**Fig. 4:**
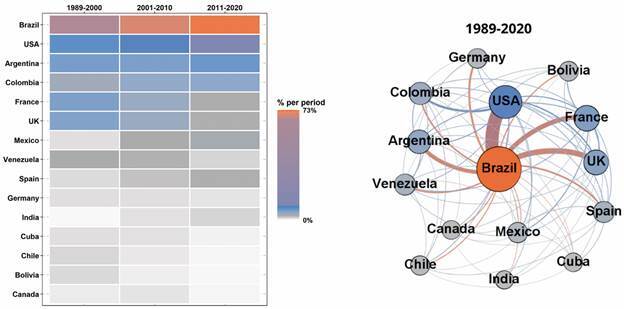
frequency heatmap and network of the top 15 countries that have published in the Memórias do Instituto Oswaldo Cruz (MIOC) (1989-2020).

The frequency heatmap (Fig. 4) indicates the percentage share of the top 15 countries that published in MIOC over three timeframes (1989-2000, 2001-2010, and 2011-2020), where the scale bar ranges from low (white) to high (orange). Changes over time are observed regarding the share of these countries in total MIOC publications. On the one hand, the top three countries increased their participation, Brazil increased its share from 67.10% in 1989-2000 to 72.45% in 2011-2020 and USA and Argentina increased their share from 7.73% and 6.29% in the first period to 10.76% and 6.96% in the last period, respectively. On the other hand, a general decrease in the publication of most other countries is verified. This includes France and the United Kingdom (UK), respectively the fifth and sixth largest participating countries concerning total MIOC publications.

The network of countries is based on author collaborations among the 15 countries presenting the highest article frequencies (Fig. 4). The nodes comprise the countries, and their size and colors are based on their weighted degree. The edge connects two countries when sharing a publication. The edge thickness is proportional to the number of collaborations. On average, each country shared at least one article with twelve other countries present in the network (average degree = 12.13). Brazil is the most central node, with the highest degree (D = 16), weighted degree (WD = 8,028), closeness centrality (CC = 1.000), betweenness centrality (BC = 0.067), and eigenvector centrality (EC = 1.000) values. The most frequent collaborations were noted between Brazil and USA (230 articles), Brazil and the UK (94 articles), and Brazil and France (78 articles). The USA and France display the second-highest degree (D = 15), closeness centrality (CC = 0.933), betweenness centrality (BC = 0.052), and eigenvector centrality (EC = 0.944) values, differing only on their weighted degree. While the USA displays the second-highest weighted degree (WD = 1,588), France exhibits the fourth (WD = 786). Excluding collaborations involving Brazil, the most relevant network collaborations were observed between Colombia and the USA (35 articles), France and UK (17 articles), the UK and the USA (17 articles), France and Mexico (14 articles), and Bolivia and France (14 articles).

From 1989 to 2020, the Oswaldo Cruz Foundation (Fiocruz) published 39.57% of the total MIOC publications. Within Fiocruz, 61.23% of its 1,777 MIOC publications are from the Oswaldo Cruz Institute (IOC), followed by the René Rachou Institute (IRR) (19.41%), and the Gonçalo Moniz Institute (IGM) (7.09%). After Fiocruz, the organisations that published in MIOC the most are the Federal University of Minas Gerais (UFMG) (7.86%), the University of São Paulo (USP) (7.78%), and the Federal University of Rio de Janeiro (UFRJ) (7.16%).

**Fig. 5:**
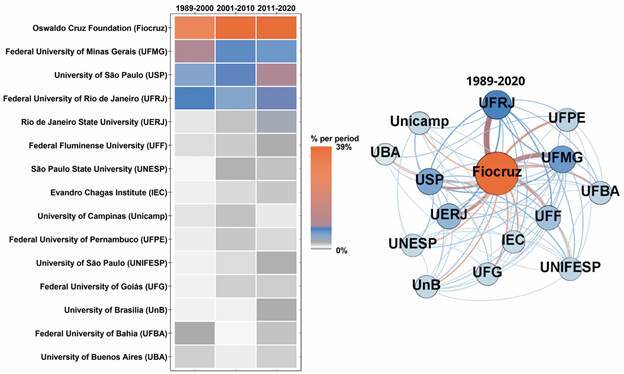
frequency heatmap and network of the top 15 organisations that have published in the Memórias do Instituto Oswaldo Cruz (MIOC) (1989-2020).

Except for the University of Buenos Aires, all the top 15 MIOC publishing organisations are from Brazil (Fig. 5). The scale bar and periods in the heatmap are the same as in the previous figure. Over time, Fiocruz continued to increase its publication share from 31.60% in the first period to 38.95% in the last. UFMG and USP, however, moved in opposite directions. While UFMG continues to have more publications than USP regarding the total period, USP ranked second in the last period concerning the ranking of organisations responsible for 9.25% of MIOC publications (UFMG comprising 6.89%). Overall, an increase in the share of most of the other organisations reported herein is noted throughout the three periods.

Regarding the organisation network (Fig. 5), the nodes represent the 15 top-publishing organisations and their size and color are proportional to their weighted degree. The edges connect organisations that have published at least one article together, and thickness reflects the total number of collaborations. On average, each organisation shared at least one article with 12 other organisations present in the network (average degree = 12.8). Fiocruz, UFMG, and USP are the most central nodes as they share the highest degree (D = 16), closeness centrality (CC = 1.000), betweenness centrality (BC = 0.042), and eigenvector centrality (EC = 1.000) values. Yet, Fiocruz exhibited the highest weighted degree (WD = 5,110), followed by UFMG (WD = 1,192) and UFRJ (WD = 1,248). The most frequent collaborations are noted between Fiocruz and UFRJ (183 articles), Fiocruz and UFMG (147 articles), and Fiocruz and the Rio de Janeiro State University (UERJ) (91 articles). Excluding Fiocruz, the most frequent collaborations take place between UFRJ and UERJ (29 articles), USP and the Federal University of São Paulo (UNIFESP) (25 articles), and UFRJ and the Federal Fluminense University (UFF) (21 articles).

**Fig. 6:**
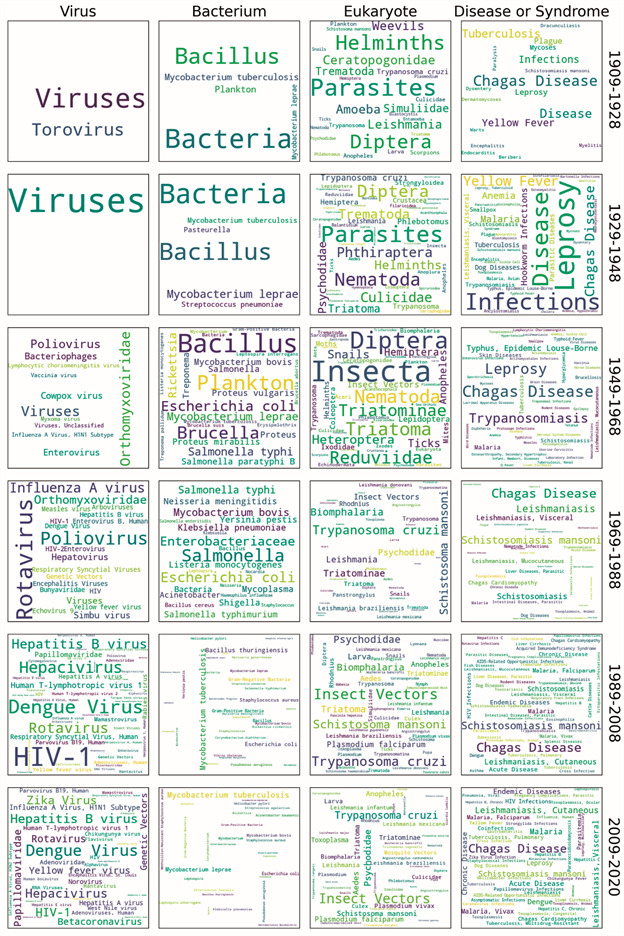
word clouds consisting of the top 50 MeSH terms (top level) within the virus, bacterium, eukaryote, and disease or syndrome semantic groups (1909-2020).

Fig. 6 indicates the word clouds of the semantic groups comprising virus, bacterium, eukaryote, and disease or syndrome, also displaying the most frequent MeSH terms (top level) of each group (maximum of 50 terms per group) throughout six timeframes: 1909-1928 (first), 1929-1948 (second), 1949-1968 (third), 1969-1988 (fourth), 1989-2008 (fifth), and 2009-2020 (sixth). Regarding the Virus group, Orthomyxoviridae was the most frequent term in the third period (three records) but does not appear from the fourth period owards (ranking third, with two records). Also during the fourth period, the term rotavirus is noted, ranking first with five records. Although its frequency increased in the fifth period to 19 records, Rotavirus ranked fourth during this period, followed by sixth in the last period. HIV-1 and dengue virus first appear in the fourth period (both ranking fifth, with one record). In the next period (1989-2008), these terms were more frequent in MIOC publications, increasing from one to 51 and 32 records, respectively, occupying the first two positions. From the fifth to the sixth period, the term dengue virus was more frequent (36 records), leading the ranking, while HIV-1 decreased its participation to 19 records, ranking fourth.

Except for the fourth period (1969-1988), *Mycobacterium tuberculosis* and *Mycobacterium leprae* appear in all periods of the Bacterium group. From the third to the fifth and sixth periods, an increased frequency was noted for *M. tuberculosis* from one to 42 and 60 records, respectively, leading the ranking in the last two periods. *M. leprae* increased from the eighth position in the fifth period (eight records), to second in the last period, with 31 records. *Escherichia coli* was also a relevant term in the bacterium group from the third period, when it first appears. Ranked third in the fifth period with 23 records, it maintained its position in the last period, although its share dropped to 15.

From the fourth period on, *Trypanosoma cruzi*, *Schistosoma mansoni*, and *Aedes* are the most relevant terms in the Eukaryote group, *i.e.*, the least generic terms with the highest frequency. Although leading the ranking in the fourth period (with 78 and 69 records, respectively), the highest frequencies for these terms were noted in the fifth period (271 and 244, respectively). In turn, a higher *Aedes* frequency was noted in the last three periods, appearing fourth in the last one, with 79 records.

Regarding the Disease or Syndrome group, Leprosy was the most frequent MeSh term in the second period (26 records) and Chagas disease the most frequent in the other periods. The leprosy term frequency was subsequently reduced, disappearing from the most frequent 50 terms during the fourth and fifth periods, and returning to the 2009-2020 rank in the eighth position (39 records). Yellow fever is relevant only in the first two periods, while Malaria comprised an important term from the second period onward, being the fourth most frequent for the 2009-2020 period, with 58 records. Appearing in all periods but the third, *Schistosomiasis mansoni* gains relevance only from the fourth period on, ranking second with 57 records. Leishmaniasis, Cutaneous appear in the fifth evaluated period, ranking third with 116 records, becoming the second most frequent term in the 2009-2020 period, with 74 records. Overall, the decrease in the number of most terms in the last period is associated to a decrease in the annual number of articles published from 2009 on. First appearing during the fourth period with two records, dengue became more relevant and ranked sixth in the 2009-2020 period, with 50 records.

**Fig. 7:**
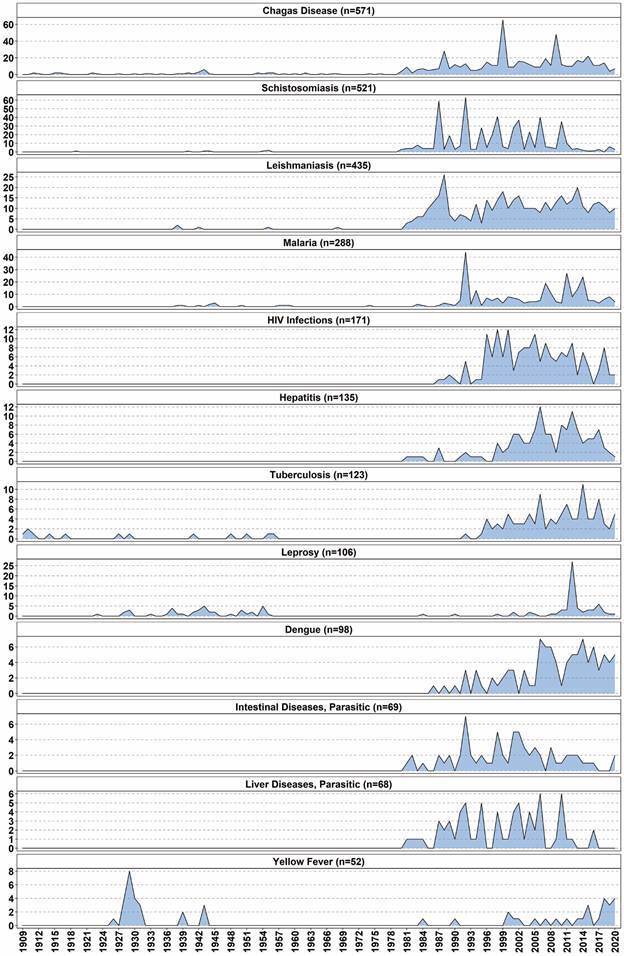
evolution over time of the top 12 diseases within the disease or syndrome semantic group (1909-2020)

The records related to the top 12 diseases within the Disease or Syndrome semantic group (1909-2020) comprised 37.53% of all articles (n = 7,027) (Fig. 7). Considering the 112 year-period and all 7,027 published articles, the most frequently cited diseases were Chagas disease (8.13% of all articles), Schistosomiasis (7.41%), and Leishmaniasis (6.19%). Between 1909 and 1980 (1,123 articles), Chagas disease presented the highest occurrence (4.54%), followed by leprosy (3.65%) and yellow fever (2.23%). In the 1981-2000 period (2,505 articles), schistosomiasis took the lead (11.66%), with Chagas disease ranking second (9.54%), followed by Leishmaniasis (7.66%). In turn, both yellow fever and leprosy reduced their participation to 0.16% and 0.12%, respectively, ranking last. In the last 20 years (2001-2020; 3,399 articles), Chagas disease ranked first (8.27%), followed by leishmaniasis (7.00%) and schistosomiasis (7.00%). Yellow fever ranked last (0.68%), following ‘Liver Diseases, Parasitic’ (0.97%), ‘Intestinal Diseases, Parasitic’ (1.06%), and leprosy (1.82%).

Most of the top 12 diseases within the Disease or Syndrome semantic group were most frequent between 2001 and 2020 (Fig. 7), with the exception of yellow fever, schistosomiasis, and ‘Liver Diseases, Parasitic’. The former was most frequent between 1909 and 1980 (25 articles), and the latter two between 1981 and 2000 (292 and 35 articles, respectively). HIV infections, hepatitis, dengue, ‘Intestinal Diseases, Parasitic’, and ‘Liver Diseases, Parasitic’ were not present in any publication between 1909 and 1980. HIV infections first appeared in MIOC in 1987, totaling 59 records in the 1981-2000 period, and 112 in the 2001-2020 period. Both hepatitis and dengue were published in over 80% between 2001 and 2020.

Table II displays the distribution of MIOC Editors-in-Chief from 1980, the top 10 MeSH Terms from the Disease or Syndrome semantic group that characterise the acting period of each Editor-in-Chief, and the MIOC Impact Factor from 1997 to 2020. The Editor-in-Chief MIOC position was formalised in January 1980, with José Coura as the first Editor-in-Chief. He held the position until December 1985 and held it again almost two decades later, from August 2001 to December 2006. The current Editor-in-Chief is Adeilton Brandão, in office since February 2019. The terms vary over time, but Chagas disease, *Schistosomiasis mansoni*, ‘Leishmaniasis Cutaneous’, and ‘Leishmaniasis, Visceral’ are relevant in all periods. The latter two, however, are more prominent from 2011, in the Ricardo Lourenço, Claude Pirmez, and Adeilton Brandão administrations. Malaria appears in all periods, gaining prominence from 2011 to 2015, along with ‘Malaria, Falciparum’ and ‘Malaria, Vivax’. This period includes Ricardo Lourenço and the beginning of the Claude Pirmez administration. Terms related to dengue, AIDS and HIV infections, Chikungunya, and Zika begin appearing in the MIOC from 1986, 1989, 2011, and 2015, respectively. Coronavirus infections appear for the first time in 2012, referring to human coronavirus NL63 and OC43 infections.^28^ This MeSH term would not appear as an index word in a MIOC publication again until 2020, with the emergence of the Coronavirus disease 2019 (COVID-19) virus.

**TABLE II:** Memórias do Instituto Oswaldo Cruz (MIOC) editors-in-chief over time, top 10 MeSH terms in the disease or syndrome semantic group (1980-2020), and MIOC impact factors (IF) (1997 to 2020)

Year	Editor-in-chief	IF	Top 10 MeSH terms
1980	José Rodrigues Coura	-	Chagas disease; schistosomiasis; leishmaniasis; leishmaniasis, visceral; Chagas cardiomyopathy; leishmaniasis, mucocutaneous; mematode infections; toxoplasmosis; intestinal diseases, parasitic; liver diseases, parasitic; rodent diseases; schistosomiasis mansoni
1981		-	
1982		-	
1983		-	
1984		-	
1985		-	
1986	Leônidas Deane	-	schistosomiasis mansoni; Chagas disease; leishmaniasis; schistosomiasis; leishmaniasis, visceral; leishmaniasis, mucocutaneous; Chagas cardiomyopathy; liver diseases, parasitic; dog diseases; malaria
1987		-	
1988		-	
1989		-	
	Eloi Garcia	-	
1990		-	
1991		-	schistosomiasis mansoni; Chagas disease; malaria, falciparum; malaria; schistosomiasis; leishmaniasis, cutaneous; cattle diseases; acute disease; babesiosis; intestinal diseases, parasitic; malaria, vivax
1992		-	
1993	Hooman Momen	-	
1994		-	
1995		-	
1996		-	Chagas disease; schistosomiasis mansoni; leishmaniasis, cutaneous; schistosomiasis; malaria; endemic diseases; acquired immunodeficiency syndrome; HIV infections; chronic disease; acute disease; AIDS-related opportunistic infections
1997		0.440	
1998		0.474	
1999		0.636	
2000		0.542	
2001	José Rodrigues Coura	0.643	schistosomiasis mansoni; Chagas disease; leishmaniasis, cutaneous; endemic diseases; acute disease; HIV infections; leishmaniasis, visceral; chronic disease; dog diseases; schistosomiasis
2002		0.635	
2003		0.688	
2004		0.740	
2005		0.847	
2006		1.208	Chagas Disease; Schistosomiasis mansoni; Acute Disease; Chronic Disease; Endemic Diseases; Leishmaniasis, Cutaneous; Leishmaniasis, Visceral; Dengue; HIV Infections; Chagas Cardiomyopathy
2007	Ricardo Lourenço de Oliveira	1.225	
2008		1.450	
2009		2.097	
2010		2.058	
2011		2.147	Chagas disease; malaria; leishmaniasis, cutaneous; malaria, vivax; malaria, falciparum; endemic diseases; leprosy; leishmaniasis, visceral; dengue; parasitaemia
2012		1.363	
2013		1.566	
2014		1.592	
2015	Claude Pirmez	1.789	
2016		2.605	Chagas disease; leishmaniasis, cutaneous; dengue; leishmaniasis, visceral; malaria; Zika virus infection; acute disease; coronavirus infections; yellow fever; endemic diseases
2017		2.833	
2018		2.368	
2019	Adeilton Alves Brandão	2.070	
2020		2.743	

The MIOC gained an IF in 1997 (IF: 0.440). From then up to 2011 (IF: 2.147), the IF exhibited a strong growth. However, in 2012 (IF: 1.363), around the middle of the Ricardo Oliveira administration, it dropped considerably, increasing to above 2.000 only in 2016 (IF: 2.605), under the Claude Pirmez administration. In 2020 (IF: 2.743), the last year of the analysed series, already under the Adeilton Brandão administration, the MIOC presented the highest IF of its history.

## DISCUSSION

The MIOC began its publication in 1909, with 16 articles on varying subjects, *e.g.*, amoeba,^29^ diptera,^30^ ^,^ ^31^ ^,^ ^32^ antibodies, antigens,^33^ bacteria,^34^ parasites,^35^ ^,^ ^36^ smallpox,^37^ tuberculosis.^38^ The first MIOC issue also brought an article by Carlos Chagas on a new human *Trypanosoma*,^39^ which would become a classic in the medical and biomedical literature, opening a line of research still in practice today at the IOC and that would become the predominant research topic disseminated by the MIOC. The first MIOC issue also published articles by other renowned researchers such as Adolpho Lutz^31^ ^,^ ^32^ ^,^ ^40^ and Arthur Neiva.^30^ ^,^ ^31^ ^,^ ^32^

In his 1909 MIOC article,^39^ Carlos Chagas describes *T. cruzi*, with the name comprising to his mentor Oswaldo Cruz,^41^ and the disease that would later bear his name, Chagas disease.^42^ In this article, Carlos Chagas reports the discovery of trypanosomes in the hindgut of blood-sucking insects, with a detailed clinical description of the acute phase of the disease, associating the infection with chronic symptoms in patients inhabiting areas where the described blood-sucking insects were endemic. The first time Carlos Chagas announced the discovery of the new trypanosome, however, was in the German journal Institut für Schiffs- und Tropenkrankheiten, while the discovery of the new disease was announced for the first time in a note in the Brazilian journal Brazil-Medico, both in 1909.^42^

The absence of MIOC publications between 1977 and 1979 is due to the military dictatorship in Brazil (1964-1985). Between 1969 and 1972, the Health Minister, Francisco de Paula da Rocha Lagoa, was also the IOC director and began to apply the administrative centralisation project devised by the military. This project involved the merger of the IOC with other organisations, forming a new organisation: the Oswaldo Cruz Institute Foundation, created in 1970 and renamed Oswaldo Cruz Foundation in 1974.^18^ For political reasons, the military regime revoked the political rights of 10 IOC researchers also in 1970 (among them Herman Lent, the head of the editorial MIOC committee), who were also compulsorily retired and forbidden to work in federally-funded government institutions. This event became known as the “Manguinhos Massacre”.^18^ The political turmoil inside and outside the walls of Fiocruz, thus, contributed to the non-publishing MIOC period from 1977 to 1979.^18^ In 1980, the MIOC formalised the Editor-in-Chief position and renews its publishing activities. José Coura then became the first Editor-in-Chief, alongside the IOC Director position until 1985.^7^

It is also from 1980 onwards that the MIOC opened up the possibility of submitting scientific studies carried out in other research institutions. Until then, the MIOC was an institutional journal dedicated exclusively to publishing articles by IOC researchers and their collaborators from other institutions.^4^ However, even after this MIOC opening, as mentioned previously, about 61% of Fiocruz’s 40% of MIOC publications between 1989 and 2020 were authored by IOC researchers. Thus, it is reasonable to assume that an important intersection between MIOC publications and research conducted at the IOC still takes place.

In 1989, another important editorial policy was changed and papers in English were prioritised.^4^ Since its first edition, papers could be published in several languages as long as there was also a Portuguese version.^5^ Similar to what was also undertaken in 1989 in the Annales de l’Institut Pasteur (the main MIOC reference since its creation), these two changes were applied for IOC to become a non-institutional and international journal.^4^ In fact, from 1980 onwards, a much higher number of articles was published annually compared to the previous period (Fig. 3), and from 1989 a variety of research organisations and countries (Figs 4-5) began publishing in the MIOC (although the most frequent were still Brazilian).

Besides José Coura, who was MIOC’s Editor-in-chief for two non-consecutive periods, MIOC has had six other Editors-in-Chief. A temporal comparison of the main MeSH terms indicated in Table II suggests that editor changes did not cause relevant changes in the published themes, as topics related to infectious and parasitic diseases remained predominant. In some periods, the themes disseminated by the MIOC accompany disease outbreaks in Brazil, such as dengue fever in the 2000s and yellow fever in the 2010s. In 2020, MIOC began publishing articles on COVID-19, following the COVID-19 pandemic, including implementing a fast track review system to prioritise these publications (MIOC: https://memorias.ioc.fiocruz.br/fast-track). However, the most frequent themes throughout the entire period are still associated to Chagas disease, schistosomiasis, leishmaniasis (Fig. 7, Table II).

It is also noteworthy that, despite the prominent role of Fiocruz during the Zika epidemic in the 2010s,^43^ only some articles were published by the MIOC on this topic. A search conducted in November 2021 at the WoS database (Supplementary data) indicated that the number of articles published on this topic worldwide increased from 21 in 2015 to about 1,500 in 2016, maintaining similar annual numbers until 2020. Of a total of 6,807 articles, 445 were published by Fiocruz researchers, one of the leading organisations in number of articles related to the Zika virus, and only 35 were published at the MIOC. Concerns that the Zika virus could become a global health threat^44^ may have directed most Fiocruz researchers to publish in foreign journals. Furthermore, although the MIOC is of significant importance in the Brazilian and Latin American scenarios, it is reasonable to assume that researchers from Fiocruz or other Brazilian organisations would prefer to publish their research in journals with greater global visibility and a much higher Impact Factor, especially when their research could be considered a scientific breakthrough. This is the case, for example, of the article published in The Lancet Infectious Diseases by Fiocruz and Brazilian researchers, where they report preliminary results of an ongoing investigation on the association between microcephaly and Zika virus infection during pregnancy.^43^

Considering the high participation of Fiocruz in MIOC publications (Fig. 5) and the greater expertise of this institution in infectious and parasitic diseases, the results of the mapping reported herein (Fig. 7, Table II) are expected. Over the years, the MIOC has established itself as an important vehicle for communicating scientific research results concerning these types of diseases,^45^ attracting researchers from all over the world. Researchers from countries that traditionally research tropical diseases, such as the USA, France, and UK, have published articles in the MIOC, often in collaboration with Brazilian researchers (Fig. 4). Concerning inter-institutional collaborations, the research collaboration network plotted herein (Fig. 5) suggests that geographical location plays an important role. Fiocruz has campuses in different Brazilian cities, and some of its main partnerships involve universities located in each city.

*In conclusion* - This study mapped articles and review articles published by the MIOC from its first issue in 1909 to 2020. This was carried out by employing bibliometrics and SNA techniques to analyse metadata from MIOC publications obtained at the SciELO, PubMed, and WoS databases. In the 1980s, the MIOC formalised the position of Editor-in-Chief, opened up to publishing research produced outside the IOC, and prioritised the English language. The results presented herein suggest that these measures were successful in transforming the MIOC into a non-institutional journal. A significant increase in annual publications is noted from the late 1980s, exceeding 100 articles for the first time in 1987. This increase partially reflects non-IOC research publication, including other scientific-technical Fiocruz units, such as IRR and IGM, as wel as Brazilian universities such as UFMG, USP, and UFRJ. The results also demonstrate important research collaboration between Fiocruz and Brazilian research organisations, and between Brazilian organisations (especially Fiocruz, which continues to lead the MIOC ranking) and foreign organisations (especially from the USA, Argentina, and Colombia).

Although the MIOC seemed to follow disease outbreaks in Brazil from time to time, the predominant research topics published in the MIOC are related to Chagas disease, schistosomiasis, and Leishmaniasis throughout the entire study period. Therefore, the editorial changes implemented from 1980 were still able to preserve the journal’s expertise in reporting biomedical research related to infectious and parasitic diseases. Based on this historical pattern, we believe that the MIOC’s publication profile is not likely to change in the coming years.

In this study, we report only what available data allows us to report. While we in no way intend to influence the direction that the MIOC will take in the future, by presenting an overview of its publication since its founding in 1909 we hope that our findings may assist MIOC Editors in decision-making and long-term planning for the journal.
