# Supplementary material for: The scientific publication of the Memórias do Instituto Oswaldo Cruz (1909-2020): a history of contribution to the biomedical sciences
Source: Mem Inst Oswaldo Cruz. 2022 Jun 13;117:e210376. doi: 10.1590/0074-02760210376 (PMC9196065; doi:10.1590/0074-02760210376)
Supplement: Supplementary file 1 [file 1678-8060-mioc-117-e210376-s.pdf]

Query:
ts = (Zika)
Refined by: DOCUMENT TYPES: (ARTICLE OR REVIEW)
Indexes = SCI-EXPANDED Timespan = 1945-2020

Number of publications per year

Year	Number of publications	Share
2020	1,607	19.63%
2019	1,509	18.44%
2018	1,536	18.77%
2017	1,422	17.37%
2016	781	9.54%
2015	21	0.26%
2014	19	0.23%
2013	4	0.05%
2012	6	0.07%
2011	2	0.02%
2010	3	0.04%
2009	4	0.05%
2008	5	0.06%
2007	1	0.01%
2006	3	0.04%
2005	3	0.04%
2004	2	0.02%
2003	3	0.04%
2002	1	0.01%
2001	3	0.04%
2000	1	0.01%
1999	2	0.02%
1998	2	0.02%
1996	3	0.04%
1994	2	0.03%
1993	2	0.03%
1992	1	0.02%
1991	1	0.02%
1988	1	0.02%
1983	2	0.03%
1982	1	0.02%
1981	1	0.02%
1979	1	0.02%
1977	2	0.03%
1973	1	0.02%
1971	1	0.02%
1969	1	0.02%
1968	1	0.02%
1965	1	0.02%
1964	2	0.03%
1954	1	0.02%
1953	2	0.03%
1952	2	0.03%

List of organisations with at least 75 publications

Organisation	Number of publications	Share
FUNDACAO OSWALDO CRUZ	445	6.54%
UNIVERSITY OF CALIFORNIA SYSTEM	371	5.45%
CENTERS FOR DISEASE CONTROL PREVENTION USA	279	4.10%
UNIVERSIDADE DE SAO PAULO	272	4.00%
UNIVERSITY OF TEXAS SYSTEM	245	3.60%
LE RESEAU INTERNATIONAL DES INSTITUTS PASTEUR RIIP	223	3.28%
STATE UNIVERSITY SYSTEM OF FLORIDA	213	3.13%
HARVARD UNIVERSITY	207	3.04%
UNIVERSITY OF TEXAS MEDICAL BRANCH GALVESTON	193	2.84%
INSTITUT NATIONAL DE LA SANTE ET DE LA RECHERCHE MEDICALE INSERM	189	2.78%
UNIVERSITY OF LONDON	184	2.70%
NATIONAL INSTITUTES OF HEALTH NIH USA	179	2.63%
CENTRE NATIONAL DE LA RECHERCHE SCIENTIFIQUE CNRS	175	2.57%
CHINESE ACADEMY OF SCIENCES	169	2.48%
INSTITUT PASTEUR PARIS	158	2.32%
JOHNS HOPKINS UNIVERSITY	154	2.26%
UNIVERSIDADE FEDERAL DO RIO DE JANEIRO	152	2.23%
UNIVERSITY OF OXFORD	149	2.19%
EMORY UNIVERSITY	147	2.16%
INSTITUT DE RECHERCHE POUR LE DEVELOPPEMENT IRD	143	2.10%
UNIVERSITY OF FLORIDA	133	1.95%
HARVARD MEDICAL SCHOOL	128	1.88%
LONDON SCHOOL OF HYGIENE TROPICAL MEDICINE	128	1.88%
NATIONAL UNIVERSITY OF SINGAPORE	120	1.76%
UNIVERSITY OF NORTH CAROLINA	117	1.72%
AIX MARSEILLE UNIVERSITE	115	1.69%
YALE UNIVERSITY	115	1.69%
NIH NATIONAL INSTITUTE OF ALLERGY INFECTIOUS DISEASES NIAID	99	1.45%
STATE UNIVERSITY OF NEW YORK SUNY SYSTEM	99	1.45%
PENNSYLVANIA COMMONWEALTH SYSTEM OF HIGHER EDUCATION PCSHE	98	1.44%
UNIVERSITY OF CALIFORNIA SAN FRANCISCO	98	1.44%
UNIVERSIDADE FEDERAL DE PERNAMBUCO	96	1.41%
UNIVERSIDADE FEDERAL DA BAHIA	92	1.35%
UNIVERSITY OF WASHINGTON	92	1.35%
UNIVERSITY OF WASHINGTON SEATTLE	92	1.35%
WORLD HEALTH ORGANIZATION	89	1.31%
UNIVERSITY OF CHINESE ACADEMY OF SCIENCES CAS	88	1.29%
STANFORD UNIVERSITY	86	1.26%
UNIVERSITE DE MONTPELLIER	85	1.25%
NANYANG TECHNOLOGICAL UNIVERSITY	84	1.23%
NANYANG TECHNOLOGICAL UNIVERSITY NATIONAL INSTITUTE OF EDUCATION NIE SINGAPORE	84	1.23%
UNIVERSIDADE FEDERAL DE MINAS GERAIS	83	1.22%
UNIVERSITY OF PISA	83	1.22%
WASHINGTON UNIVERSITY WUSTL	83	1.22%
GERMAN CENTER FOR INFECTION RESEARCH	81	1.19%
MINIST HLTH	78	1.15%
UNIVERSITY OF MIAMI	77	1.13%
UNIVERSITY OF NORTH CAROLINA CHAPEL HILL	77	1.13%
UNIVERSITY SYSTEM OF GEORGIA	77	1.13%
UNIVERSIDADE FEDERAL DE SAO PAULO UNIFESP	75	1.10%

List of journals with at least 25 publications

Journal	Number of publications	Share
PLOS NEGLECTED TROPICAL DISEASES	305	4.48%
SCIENTIFIC REPORTS	225	3.31%
VIRUSES BASEL	197	2.89%
PLOS ONE	182	2.67%
JOURNAL OF VIROLOGY	117	1.72%
PARASITES VECTORS	110	1.62%
EMERGING INFECTIOUS DISEASES	98	1.44%
ANTIVIRAL RESEARCH	97	1.43%
AMERICAN JOURNAL OF TROPICAL MEDICINE AND HYGIENE	88	1.29%
NATURE COMMUNICATIONS	86	1.26%
FRONTIERS IN MICROBIOLOGY	85	1.25%
JOURNAL OF MEDICAL ENTOMOLOGY	76	1.12%
JOURNAL OF INFECTIOUS DISEASES	73	1.07%
FRONTIERS IN IMMUNOLOGY	64	0.94%
MMWR MORBIDITY AND MORTALITY WEEKLY REPORT	63	0.93%
ACTA TROPICA	57	0.84%
EMERGING MICROBES INFECTIONS	57	0.84%
PLOS PATHOGENS	57	0.84%
BMC INFECTIOUS DISEASES	54	0.79%
EUROSURVEILLANCE	53	0.78%
CELL REPORTS	50	0.74%
INTERNATIONAL JOURNAL OF ENVIRONMENTAL RESEARCH AND PUBLIC HEALTH	44	0.65%
INTERNATIONAL JOURNAL OF INFECTIOUS DISEASES	43	0.63%
VIROLOGY	43	0.63%
PROCEEDINGS OF THE NATIONAL ACADEMY OF SCIENCES OF THE UNITED STATES OF AMERICA	42	0.62%
VACCINE	42	0.62%
MBIO	41	0.60%
JOURNAL OF MEDICAL VIROLOGY	38	0.56%
TRANSFUSION	38	0.56%
INTERNATIONAL JOURNAL OF MOLECULAR SCIENCES	37	0.54%
MEMORIAS DO INSTITUTO OSWALDO CRUZ	35	0.51%
CELL HOST MICROBE	34	0.50%
JOURNAL OF THE AMERICAN MOSQUITO CONTROL ASSOCIATION	32	0.47%
VIRUS RESEARCH	31	0.46%
JOURNAL OF CLINICAL VIROLOGY	30	0.44%
JOURNAL OF GENERAL VIROLOGY	29	0.43%
INFECTION GENETICS AND EVOLUTION	27	0.40%
LANCET INFECTIOUS DISEASES	27	0.40%
FRONTIERS IN CELLULAR AND INFECTION MICROBIOLOGY	26	0.38%
NATURE MICROBIOLOGY	26	0.38%
VIROLOGY JOURNAL	26	0.38%
ASIAN PACIFIC JOURNAL OF TROPICAL MEDICINE	25	0.37%
CURRENT OPINION IN VIROLOGY	25	0.37%
JOURNAL OF CLINICAL MICROBIOLOGY	25	0.37%
VACCINES	25	0.37%
EBIOMEDICINE	24	0.35%
ENVIRONMENTAL SCIENCE AND POLLUTION RESEARCH	24	0.35%
PATHOGENS	24	0.35%
SCIENCE	24	0.35%
CLINICAL INFECTIOUS DISEASES	23	0.34%
